# GPs’ and child and adolescent psychiatry specialists’ experiences of joint consultations in the GP’s office: a qualitative study

**DOI:** 10.1186/s13104-017-2766-7

**Published:** 2017-09-07

**Authors:** Tori Guldahl Seierstad, Mette Brekke, Ingun Toftemo, Ole Rikard Haavet

**Affiliations:** Department of General Practice, Institute of Health and Society, University of Oslo, Oslo, Norway

**Keywords:** Children, Adolescents, Mental health, General practice, Collaboration, Joint consultation

## Abstract

**Background:**

The study is an exploration of a joint consultation model, a collaboration between general practitioners (GPs) and specialists from child and adolescent mental health services (CAMHS) in Lillehammer, Norway.

**Methods:**

A qualitative study based on two focus group interviews, one with participating GPs and one with participating specialists from the local CAMHS. Participants were five GPs, with work experience varying from 6 months to 20 years (four of them specialists in general medicine) and two CAMHS specialists—a psychiatrist and a psychologist—both with more than 20 years of experience.

**Results:**

The focus group discussions revealed that both GPs and CAMHS specialists saw the joint consultations as a good teaching method for improving GPs’ skills in child and adolescent psychiatry. Both groups believed that this low-threshold service benefits the patients and that the joint consultation is especially suited to sort problems and determine the level of help required.

**Conclusions:**

The GPs and CAMHS specialists shared the impression that the collaboration model is beneficial for both patients and health care providers. Close collaboration with primary health care is recommended in the guidelines for child and adolescent psychiatry outpatient clinics. We suggest that the joint consultation model could be a good way for GPs and CAMHS specialists to collaborate.

## Background

GPs do have a role in child and adolescent mental health care [1–4]. It is, however, shown that GPs’ skills in child and adolescent psychiatry could be improved [1, 3, 5–11]. Hafting has shown that an interested GP is in a good position to provide services to children and adolescents with mental health problems, but that many GPs believe that they lack specific knowledge in child and adolescent psychiatry [1]. Zwaanswijk found that approximately 80% of children and adolescents with psychological problems had seen their GP within the preceding year, but GPs’ identification of psychological problems was limited [6]. These findings are consistent with other studies [5, 12]. Alexander and Heikkinen have both displayed that GPs perceive their skills in child psychiatry as somewhat inadequate [7, 8]. The need for increased knowledge and skills among the GPs in this field is addressed several in other papers [3, 9, 11, 13]. It is therefore important to gain more knowledge as to how GPs’ competence and skills can be further developed. Generally, collaborative programmes seem to be more effective than more theoretical educational programmes [14–20], in line with theories of experiential learning [21]. A Cochrane review examining interventions to change outpatient referral rates or improve outpatient referral appropriateness found that active local educational interventions involving secondary care specialists and structured referral sheets are the only interventions that lower referral rates, based on current evidence [22].

There are various types of on-site mental health interventions, and in the literature, it is not always clear exactly what is included [23]. Different types of collaboration have been described, ranging from a consultative role only for a psychiatrist, [24, 25] to consultation-liaison psychiatry [26–29], specialist clinics in a primary care setting [30], telepsychiatry [29, 31] or collaborative care with various mental health care providers [20, 32, 33]. We have found joint consultations with GPs described in adult psychiatry [34–36] and in paediatrics [16, 17], but we have found no studies that specifically describe joint consultation in a general practice with child and adolescent psychiatry specialists. In an adult psychiatry setting, Saillant describes collaboration between primary care physicians in training and a psychiatrist, and suggests that joint consultation is an efficient way to transfer skills and build confidence among primary care physicians in their care of patients with mental health problems [34]. Mouland describes a model in which adult patients often obtained sufficient help from one joint consultation with the GP and the psychiatrist [35]. Macaulay shows that joint clinics with GPs and pediatric specialists can improve clinical knowledge and skills for both [16]. Similar results are described in an evaluation of a model where hospital paediatricians and GPs participate in joint clinics and multidisciplinary team meetings in GP practices [17]. In a pilot study from 1997, a primary mental health care worker provided expert support and advice and specialist consultations to GPs in Portsmouth [26]. This programme resulted in fewer referrals to child and adolescent mental health services in the intervention group, and the referrals that did occur were more likely to be assessed as appropriate.

Norwegian guidelines for mental health services for children and adolescents state that outpatient clinics should collaborate closely with primary health care givers, including GPs [37]. A qualitative study from 2011 that examines the collaboration between GPs and mental health care professionals in Norway suggests that face-to-face contact between GPs and mental health professionals seems to be superior to written or telephonic contact in easing collaboration [38].

Since joint consultations with GPs have been suggested to be useful in both adult psychiatry [34, 35] and paediatrics [16, 17], it is interesting to see how a similar approach will work in child and adolescent psychiatry. This paper describes such a model: During the period 2008–2014, the GPs in one group practice in Lillehammer, Norway practiced joint consultations with professionals from the local child and adolescent psychiatry clinic. Our study is an exploration of the experiences of this collaboration among participating GPs and specialists from the local CAMHS Outpatient Clinic. Our aim has been to evaluate possible benefits and downsides of the collaboration, which could be useful to other GPs and CAMHSs who consider implementing something similar.

## Methods

### Settings

The collaboration incorporates joint consultations at the GP’s office at Svingen Health Centre. When there is concern about the psychological health of a child or adolescent, the GP can make an appointment with one of the participating child psychiatry specialists. Every fortnight (except during school holidays) the GPs have two available 1-h appointments. If it is obvious that the child needs specialist psychiatric care, the GP can still refer directly to the specialist clinic. The collaboration began in August 2008 as an experiment. When the focus group discussions took place in October and November 2014, 100 joint consultations had been held, with 76 children and adolescents. We encourage both parents to come with the child. The GP and the child psychiatry specialist usually have a brief talk before the GP fetches the family from the waiting room. The GP starts by presenting the visiting specialist to the parents and child and informing them about the collaboration. The GP then briefly introduces the background for the consultation and invites the parents and the child if they have any questions or comments. The CAMHS specialist usually takes the lead in establishing contact with the child and the parents. From time to time, the GP makes a comment or is consulted by the CAMHS specialist, especially where somatic symptoms are involved. The primary function of the joint consultation is to decide whether the child requires a referral or if the problem can be handled in primary care—whether by the GP, the school psychology services, the family team at the child health centre, the child welfare services, or others.

During these consultations, the CAMHS specialist acts as a consultant. The documentation is the GP’s responsibility; nothing is registered at the CAMHS clinic. If the patient is not referred, the GP is responsible for follow-up and referral to other services as needed. Sometimes a new joint consultation is established; sometimes the GP who had decided that a referral was unnecessary later decides to refer the child after all.

### Design

Two focus group discussions based on semi-structured interviews, one with the participating GPs (October 2014) and one with the participating CAMHS specialists (November 2014).

### Participants

Five GPs working in the same practice; their experience in general practice varied from 6 months to 20 years, with a median of 12 years. Two CAMHS specialists—one psychiatrist and one psychologist—both had more than 20 years of experience and worked together.

### Data sampling

The participants gave their informed consent to take part in semi-structured interviews conducted by the third author, a GP from another health centre in town with experience from qualitative research. The first author was present, but did not participate in the discussions. An interview guide was used to generate discussion. Topics included a general impression of the collaboration, whether the participants had learned something from the collaboration, examples of successful and unsuccessful consultations, suitable and unsuitable issues for collaboration, and ways of making the collaboration work in practice. Our aim was to evaluate possible benefits and downsides of the collaboration. TS prepared a question guide to shed light on these questions. All authors commented on the guide and the question guide was finalized by TS and IT. The following questions were used as guidance for the semi-structured interviews (Table 1).

**Table 1:** Interview guide

	Question	Comment
1	How have you experienced having joint consultations with CAMHS specialists in your office? / How have you experienced visiting the GP’s office for the joint consultations?	Focus on the emotional aspect of having a consultation with another professional. Has it felt difficult or uncomfortable? If yes, why? If no, why?
2	Have you learnt something from the joint consultations? If yes, can you describe what you have learnt. Do you think the CAMHS specialists/the GPs have learnt anything? If yes, what?	
3	Can you tell us about an especially successful consultation? When the child wasn’t referred? When the child was referred? How do you think the family felt about the consultation?	Not necessarily all examples from everyone, but try to get all alternatives covered
4	Can you tell us about a not so successful consultation? When the child wasn’t referred? When the child was referred? How do you think the family felt about the consultation?	Not necessarily all examples from everyone, but try to get all alternatives covered
5	For what kind of problems do you find the joint consultation especially useful?	
6	Are there problems that are not suitable for joint consultations?	
7	What factors are important for such a collaboration to work?	
8	Would you recommend joint consultations to other GPs/CAMHS clinics?	
9	Anything else you want to mention?	

### Analysis

Interviews were recorded digitally and transcribed verbatim. The transcripts were imported into the software package NVivo 10 [39]. All authors read through the interviews by themselves. Interviews were then analysed via systematic text condensation by the first author, as described by Malterud [40]. During the process of coding for units of meaning TS and IT discussed and agreed on the different codes. Analysis followed four steps: (i) listening to sound tracks and reading transcripts to obtain an overall impression; (ii) identifying and coding for units of meaning representing different aspects of the collaboration in question; (iii) condensing and summarizing contents of each coded group; and (iv) based on these groupings, describing the participants’ experiences and opinions about the collaboration model via the thematic categorization as presented below.

## Results

Between August 2008 and August 2014, a total of 76 patients were seen in 100 consultations. One or both parents attended most consultations, another carer attended four consultations, six adolescents came alone, and in eight consultations one or both parents came without their child. Table 2 shows the gender and age at first consultation and Table 3 shows the age distribution of all consultations.

**Table 2:** 76 children seen in joint consultations—gender and age

	Girls	Boys
Gender	35 (46%)	41 (54%)
Age at first consultation (years)		
Mean	10.5	9.5
Minimum–maximum	0–18	2–16

**Table 3:** Age of children seen in 100 joint consultations

Age group (years)	Percentage of consultations (%)
<5	10
6–9	30
10–13	40
14–17	19
>18	1

The median time between presenting the problem to the GP and participating in the joint consultation was 20 days; 38% had their appointment within 2 weeks, 24% of patients had to wait more than 4 weeks (often during holiday times). Of the 76 children, 11 were referred to the CAMHS at the initial joint consultation, and 11 were referred later; thus 71% were not referred.

Behavioural problems and concerns about hyperkinetic disorder were the most common presenting problem, each present in 19% of the consultations. Other common issues were anxiety (15%); depression (12%); psychiatric or drug-related problems in the family (12%); reaction to stress (11%—often parents’ divorce); non-attendance at school (10%); and psychosocial problems at school, including bullying (10%). Issues present in less than 10% of the consultations were, from most to least frequent, need for consultation about a known diagnosis, tics, psychosomatic symptoms (including enuresis and encopresis), learning difficulties, worries about autism spectrum disorder, sleeping problems, eating disorders, self-harming, and concerns about early mother–child interaction.

Analysis revealed the same main themes in both focus groups: The GPs and CAMHS specialists agreed that children and families receive help from the consultations and that there are learning effects for the GPs and benefits for the CAMHS. The value of the two groups of health care providers having the opportunity to meet and talk person-to-person was emphasized. Downsides of the collaboration were also reflected upon, along with practical issues like when to use the joint consultation and how to make it work. These themes are presented below.

### Help for children and their families

In both focus groups, the collaboration was viewed as a low-threshold service, where help can be given close to the patient.

GP: *It’s like an ordinary consultation, right, just with a hired expert.*

The interviewees reported that children and parents find their GP’s office a safe place to come, whereas going to the CAMHS at the hospital can seem frightening to some of them.

GP: *I believe the fact that we are confident in* [the specialists]*, affects the patients. They know us, right, and they see that we trust and like the company of the person from the child and adolescent psychiatry clinic, which makes it easier for them to relax and open up too, it’s astonishing, you know, to see how people are able to open up faced with a person they never met before. It’s pretty good. And there, I believe we have kind of a catalyst role, just by being there.*

Both focus groups reported that many families are happy that things can be solved at the GP’s practice, so they need not be referred and undergo what is often a time-consuming process.

GP: *‘Cause they had the abilities in the family to handle this. Right? So that was very good. The alternative would have been to refer. And that would have been a lot more hassle.*

### GPs’ learning effects

The GPs said that they had learned a great deal from seeing how the specialists talked with the children—that they could be quite direct with them.

GP: *The short version is that they are quite direct. (…) They call a spade a spade.*

The GPs said that their diagnostic skills had improved and that they had learned how to explore for different problems, so they could better sort which patients to refer and which not to refer. The specialists agreed with the GPs self-evaluations.

CAMHS: *Learning to sift. It’s obvious that we achieve that with Svingen Health Centre.*

The GPs said they had also received concrete advice and learned strategies to use when treating such conditions as phobias and sleeping disorders.

GP: *Next time I can try that for myself.*

### Benefits for the child and adolescent psychiatry clinic

The GPs were not sure what the specialists can learn from them, other than gaining insight into the way a GP works, seeing a broader range of patients, and obtaining some somatic knowledge. As the psychologist confirms:

CAMHS: *I think it’s been a benefit to the clinic to have somatic medicine tied closer to us. Probably it’s been useful to me, who doesn’t have a medical education.*

The GPs said they think the CAMHS specialists enjoy this way of working, which the specialists confirm.

CAMHS: *We meet the GP as consultants. That is to say, we’re not responsible for the things that are brought up, all the documentation. In a way, taking care of the patients, that’s the GPs’ responsibility.*

The specialists also found that there were now fewer referrals from Svingen Health Centre, and that the referrals that did come, were more appropriate.

CAMHS: *And they get it, the GPs at Svingen. Later, with a similar case, you don’t refer this. You can handle it yourself.”*

### The value of meeting each other

The GPs stressed the importance of meeting the CAMHS specialists in person, for learning effects, for the good of the specific patient, and for an opportunity for a short, informal consultation about other patients.

GP: *There’s so little contact between us and the specialist health services, face to face. Right? We never meet. So, having such meeting points is important.*

The CAMHS specialists also agreed that meeting their colleagues in primary health care was important, for both sets of health care workers and for the patients.

CAMHS: *The function of getting together early and looking at things together…An unbeatable team, when the GP and someone from the child psychiatry meet, I think, having some experience, there’s a lot of power in that meeting.*

### Is there no downside?

Both GPs and specialists mentioned the possibility that the joint consultation could lead GPs to cut down on referrals of children and adolescents who should have been referred. The GPs were aware that one of the clinic’s reasons for suggesting the collaboration was to reduce the number of referrals. Some of the GPs were concerned that they could lead the consultation in the wrong direction and miss important information.

GP: *And then I’ve thought sometimes, if I’ve been thinking wrong to start with and lead things in a wrong direction, then we miss.*

Some GPs expressed discomfort with the responsibility they were expected to assume after they had decided not to refer.

GP: *I’ve been thinking of the first consultation, the first joint consultation, that, well, I felt I got a little too much responsibility.*

At the same time, they went on to explain how these issues were counteracted so as not to constitute major problems. The CAMHS specialists pointed to their prioritizing guidelines, which state that the more severe disorders are their responsibility. The GPs stressed that by following up on their patients, they’ll be able to see if other measures need to be taken.

GP: *Even if* [the psychologist] *said not to refer, you think, yes, well, it’s your right to refer anyway.*

### When to use the joint consultation?

The GPs and the specialists agreed that a joint consultation is not suitable if the child’s parents are in conflict and some GPs said that they would be reluctant to participate if severe psychiatric symptoms were in question.

CAMHS: *The worst is when children experience their parents screwing up, to put it simply. I don’t want to witness that.*

Everyone saw the joint consultations as useful for sorting the problems. Is this a normal reaction? Should it be referred to the pedagogic–psychological service? Can the GP handle this, or should the CAMHS take over?

CAMHS: *I think that to take part and sort, trying to separate the wheat from the chaff, there, I believe, our two disciplines have much to gain from each other.*

Both groups also mentioned concrete problems like phobias and encopresis as suitable issues for joint consultation.

CAMHS: *Everything to do with poop, pee, head, stomach, everything to do with psychosomatics for example, is brilliant* [for joint consultation].

### How to make it work in practice?

The GPs mentioned several practical issues that were necessary to make this work in a busy practice. There must be fixed, sufficiently frequent appointments that are easy for the GPs to book. Both groups also stated that a successful collaboration depends a great deal on the specialists; not everyone would be comfortable or do a good job in such a setting.

GP: *But I believe the main success factor is personal. One must bear that in mind, right?* [They are] *confident, very experienced specialists who dare go straight to the core of the problems, you know, so that people feel that what’s happening is important, and that you get help.*

## Discussion

### Statement of principal findings

We found that the specialists and the GPs shared the impression that the collaboration is useful; its benefits clearly outnumbering negative effects. They described benefits for the patients, the GPs, and the CAMHS. Both groups expressed the belief that patients find it easier to come to the GP than to the specialist clinic, that it feels safe, that it is a low-threshold service, and that many families are happy not to be referred. One model for collaboration is “meeting between experts” and another is “transference of competence from one level to another”. We find that the experiences from the present project are more like the last. Although the CAHMS specialists say that they benefit from coming closer to somatic medicine, both GPs and CAMHS specialists describe more learning effects for the GPs than for the CAMHS specialists. GPs reported themselves as being more confident now when talking to children and adolescents about difficult issues and that their diagnostic skills in child and adolescent psychiatry had improved. The psychiatrist and the psychologist have the impression that there are now fewer and more adequate referrals. Both GPs and CAMHS specialists are aware of the fact that one motivation of CAMHS for entering the collaboration is a reduction in the number of referrals, and that this could lead to patients not receiving the help they need. To counteract this potential problem, the GPs say that they can detect through follow-up if the situation is not improving, and then take action. The CAMHS specialists point to the priority guidelines to which they should adhere. The two groups agreed upon the issues that are more suitable for these joint consultations, and that it is especially useful for sorting the wheat from the chaff. They also agreed that the mental health specialists must be confident and experienced for the collaboration to be successful.

### Strength and weaknesses of the study

In the GP focus group, one of the GPs had had only one joint consultation and participated little in the focus group discussion; the other four were all active during the discussion. The youngest of the GPs had approximately 6 months’ experience and was substituting during that time for the first author, who had participated in the consultation project since its start in 2008 and was an observer to the discussion. A possible weakness of this study is that one the GPs who had taken part in the collaboration from the beginning (i.e. the first author) did not participate in the discussion. Another problem is her being biased from being a part of the collaboration, and analysing the discussion between her colleagues. To minimize this potential bias all four authors have read the interviews, and the coding was discussed between them during the analysis. The interview was performed by a GP from a neighbouring health centre that is not part of the collaboration in question and who is also a researcher with experience from focus group interviews.

There were only two CAMHS specialists involved in the collaboration. Although focus groups usually consist of more than two persons, we chose to do a group interview also with the psychiatrist and the psychologist, reckoning that this after all would make the discussion more comparable to the one in the GP group than if we had done individual interviews. A better option might have been to divide the GP group in two, to make the groups more comparable in size, or to conduct individual interviews with all participants. Still, the dynamics in the groups were similar and the results were largely coincidental.

A limitation of focus groups is the extent to which participants (usually strangers) are willing to reveal their feelings and opinions to the rest of the group and the facilitator. In this study, the participants in both groups were colleagues who knew each other well. The first author was present and observed both interviews, and had a clear impression that the participants were open and talked freely about the issues that arose.

The transferability of our findings may be limited by the fact that only one health centre and one speciality clinic was involved in the collaboration. As the participants argue, the success of this model depends largely on personal factors—on the participants being comfortable with an interdisciplinary collaboration. Further research on a broader and more representative group of GPs and child and adolescent psychiatry clinics is needed to determine if this would be a suitable collaboration in other contexts.

The health care providers argue that the collaboration is helpful for the patients, but the researchers did not have the resources to interview children and their families. This addition to the study would have provided greater insight into the experiences of the patients.

### Findings in relation to other studies

There are some examples of consultation–liaison psychiatry for children and adolescents, the most similar to ours being a pilot study by Neira-Munoz, wherein GPs were assisted by a primary mental health care worker who provided expert support and advice and specialist consultations [26]. Similar to the impression in our study, this programme resulted in fewer referrals to child and adolescent mental health services by the GPs in the intervention group, and the referrals were more likely to be assessed as appropriate. A Canadian study describes how the integration of mental health teams in a primary care setting has improved access to mental health care for children and expanded the capacity of primary care physicians to deliver mental health care [32]. As in our study, they mention the advantages of a low-threshold service and argue that secondary and tertiary mental health service efficiency is improved because of better triaging of patients in primary care before referral. This project involved several health professions and had central management, as opposed to our small-scale collaboration with virtually no administration. Studies from paediatrics and adult psychiatry support the findings from our study. Both Macaulay and Montgomery-Taylor find that a model with joint consultations with GPs and paediatricians provides learning for the participating GPs [16, 17]. Mouland states that patients often receive sufficient help from one joint consultation with the GP and the psychiatrist [20] and Saillant’s study suggests that joint consultation is an effective way of transferring skills and building confidence in primary care physicians regarding care of patients with mental health problems [21]. Saillant discusses the limitation that they, as we, were unable to explore the experiences of the patients. This would be a key area for further research.

In general, the value of meeting face to face is a common finding in studies about collaboration between primary and secondary health care professionals, a finding that is in agreement with our results. Compared to other examples, our model involves few persons and little administration, which makes it easy to implement.

## Conclusions

There are several collaboration models involving GPs and other mental health professionals, but we have found no other studies evaluating joint consultations between GPs and child and adolescent psychiatry specialists. Therefore, although our study is a small-scale collaboration, we think it is an interesting addition to what is already published, and we hope it can encourage others to new collaborations and further research. Norwegian guidelines for mental health services for children and adolescents state that outpatient clinics should collaborate closely with primary health care, and joint consultation could be an effective way of doing so.

## Abbreviations

GP: general practitioner
CAMHS: child and adolescent mental health services
